# Integration event induced changes in recombinant protein productivity in *Pichia pastoris* discovered by whole genome sequencing and derived vector optimization

**DOI:** 10.1186/s12934-016-0486-7

**Published:** 2016-05-20

**Authors:** Jan-Philipp Schwarzhans, Daniel Wibberg, Anika Winkler, Tobias Luttermann, Jörn Kalinowski, Karl Friehs

**Affiliations:** Fermentation Engineering, Bielefeld University, Universitätsstr. 25, 33615 Bielefeld, Germany; Microbial Genomics and Biotechnology, Center for Biotechnology (CeBiTec), Bielefeld University, Universitätsstr. 27, 33615 Bielefeld, Germany; Genome Research of Industrial Microorganisms, CeBiTec, Bielefeld University, Universitätsstr. 27, 33615 Bielefeld, Germany; CeBiTec, Bielefeld University, Universitätsstraße 25, 33615 Bielefeld, Germany

**Keywords:** *Pichia pastoris*, Recombinant protein production, *AOX1* promoter, Genome sequencing, Insertion locus, Non-conventional yeast, Expression cassette orientation, False-positive

## Abstract

**Background:**

The classic *AOX1* replacement approach is still one of the most often used techniques for expression of recombinant proteins in the methylotrophic yeast *Pichia pastoris*. Although this approach is largely successful, it frequently delivers clones with unpredicted production characteristics and a work-intense screening process is required to find the strain with desired productivity.

**Results:**

In this project 845 *P. pastoris* clones, transformed with a GFP expression cassette, were analyzed for their methanol-utilization (Mut)-phenotypes, GFP gene expression levels and gene copy numbers. Several groups of strains with irregular features were identified. Such features include GFP expression that is markedly higher or lower than expected based on gene copy number as well as strains that grew under selective conditions but where the GFP gene cassette and its expression could not be detected. From these classes of strains 31 characteristic clones were selected and their genomes sequenced. By correlating the assembled genome data with the experimental phenotypes novel insights were obtained. These comprise a clear connection between productivity and cassette-to-cassette orientation in the genome, the occurrence of false-positive clones due to a secondary recombination event, and lower total productivity due to the presence of untransformed cells within the isolates were discovered. To cope with some of these problems, the original vector was optimized by replacing the *AOX1* terminator, preventing the occurrence of false-positive clones due to the secondary recombination event.

**Conclusions:**

Standard methods for transformation of *P. pastoris* led to a multitude of unintended and sometimes detrimental integration events, lowering total productivity. By documenting the connections between productivity and integration event we obtained a deeper understanding of the genetics of mutation in *P. pastoris*. These findings and the derived improved mutagenesis and transformation procedures and tools will help other scientists working on recombinant protein production in *P. pastoris* and similar non-conventional yeasts.

**Electronic supplementary material:**

The online version of this article (doi:10.1186/s12934-016-0486-7) contains supplementary material, which is available to authorized users.

## Background

*Pichia* (*Komagatella*) *pastoris* is a non-conventional methylotrophic yeast that is widely used as a host for recombinant protein production [1, 2]. Its capability to perform post-translational modifications such as disulphide isomerization or glycosylation, an efficient secretion apparatus and the relative ease of reaching high dry cell weights >100 g/L during bioreactor fermentation make this eukaryote a popular choice for protein expression in industry as well as in research [3–5]. Over 500 proteins, from industrial enzymes to biopharmaceuticals, have been expressed in *P. pastoris* [6]. A growing number of commercial products have reached the market in recent years [7]. Among them are the FDA-approved drugs Kalbitor^®^ and Jetrea^®^, a kallikrein inhibitor and an aglycosylated protease, respectively [8].

The most common approach for heterologous protein expression in *P. pastoris* is the insertion of the target gene into the genome under the control of the *AOX1* (alcohol oxidase 1) promoter (p*AOX1*). This approach offers tight regulation and a very strong, methanol-inducible expression [6]. Two different modes of homologous recombination-mediated insertion are typically used (i) ends–in insertion leads to additive insertion of the target gene and (ii) ends–out insertion facilitates the replacement of a genomic region, most commonly the native *AOX1* gene [9]. Knock-out of *AOX1* leads to the Mut^S^-phenotype (methanol utilization slow), since only the lesser transcribed *AOX2* gene remains. Clones with an additive insertion retain the native phenotype Mut^+^ (methanol utilization plus, full growth on methanol). The optimal phenotype for a given application can differ, with Mut^S^-strains exhibiting higher productivity than Mut^+^-clones in some cases [10, 11]. Recently much progress has been made in understanding the regulation of p*AOX1* as well as creating novel synthetic variants with improved characteristics, underlining that the importance of the promoter still holds [12–17].

A frequently encountered problem during generation of *P. pastoris* clones via homology-mediated integration of the expression cassette is the low targeting efficiency, being as low as <1 % in certain cases like the mannosyltransferase *OCH1* [18]. In addition, an off-target insertion can lead to the disruption of a gene and potentially affect production characteristics. Different techniques have been proposed to improve the targeting efficiency, e.g. preventing random insertion due to non-homologous end-joining (NHEJ) via deletion of a *KU70* homologue or increasing the genetic redundancy [18, 19]. While these methods help to reduce the number of untargeted insertions, they are better suited for genetic engineering studies rather than the generation of a production strain. Scientists working with *P. pastoris* are faced with the task of identifying the optimal producer from a diverse group of clones with varying production characteristics. Similar problems have been reported for other non-conventional yeasts that are often used for recombinant protein expression like *Hansenula polymorpha*, *Yarrowia lipolytica* and *Kluyveromyces lactis* [20–22].

The publicly available genome sequences for the most commonly used *P. pastoris* strains CBS 7435 [23] and GS115 [24] gave rise to multiple genome-scale experiments, most of which focused on better understanding metabolic pathways in order to improve yields in recombinant protein production [25–28]. However, to present no study has been published that investigates the effects of random insertions on the productivity in *P. pastoris* as well as the integration events on the genome scale. In essence a sort of “black box” is present during transformation of *P. pastoris* and it is uncertain if the clone with the desired characteristics will be generated. Unknown events during integration of the expression cassette can lead to drastically different production characteristics of clones from one transformation experiment.

For researchers working with *P. pastoris*, or similar non-conventional yeasts, it would be of great value to gain insights into what might cause unexpected expression levels. By correlating an insertion event seen on the genome with the production characteristics their interaction can be determined. Once these events are known steps can be taken to e.g. optimize vectors to prevent particular integration events.

Using methods previously described and established specifically for *P. pastoris*, a library of 845 clones was characterized for their expression levels and gene copy numbers (GCN) of GFP*uv* (cycle-3-GFP) [29] as well as their Mut-phenotypes [30–33]. Based on these characteristics the clones were grouped and the 31 most outstanding ones selected for genome sequencing. By correlating experimental and genome data novel insights into the integration event and its effect on productivity were discovered and the original vector optimized.

## Results and discussion

### Characterization and grouping of pAHBgl-GFP *P. pastoris* clones

In total, 845 *P. pastoris* clones transformed with the GFP expression cassette were characterized for their Mut-phenotypes, GFP gene expressions and GCN. The intent of the transformation strategy used in our study was to replace the native *AOX1* gene with a single copy of the GFP expression cassette. Therefore a “regular” clone should have the Mut^S^ phenotype as well as GCN and GFP expression level of around 1. Overall, 347 out of all 845 clones fall into this category, accounting for approximately 41 % of all clones. This targeting efficiency for *AOX1* is above previously reported values of around 25 % [34, 35]. It has to be considered that in the present study a different histidine auxotroph strain, CBS7435 (Δ*HIS4*) with a fully deleted *HIS4* gene was used [19]. Thereby the background of spontaneous histidine prototrophy conversion clones found in GS115, in which histidine-auxotrophy is mediated by a single nucleotide polymorphism (c.1669C > T resulting in p.557Arg > Cys), is eliminated and the proportion of Mut^S^ strains increased. Strains with the Mut^+^ phenotype can exhibit negative traits due to illegitimate recombination of the expression cassette into the genome and require more methanol for continuous induction. Nevertheless, they might present suitable hosts for protein expression if adjusting process parameters accordingly [11]. Hence, Mut^+^ strains that otherwise displayed the same features as regular clones can also be considered suitable for most applications. To this end, they were added to the “regular” clones in this study.

All strains not falling into either of these categories were designated as “irregular”. They displayed certain properties that were not expected based on the transformation modus. Table 1 shows the distribution of strains based on these criteria and their Mut-phenotypes. While about a quarter of all clones exhibited irregular features only five of these were Mut^S^ strains, underlining the higher genetic variance of Mut^+^ clones. A total of 45 multi-copy clones (GCN ≥1.5) were found, accounting for ca. 5 % of all clones. Among them seven “jackpot” strains with a GCN >10 are present. All our subsequent analysis concentrated on the irregular clones, in order to elucidate the genetic cause of their aberrant properties.

**Table 1:** Distribution of all 845 *P. pastoris* clones, transformed with the GFP expression cassette, based on “regular” and “irregular” properties as well as the Mut-phenotype

Group	No. of clones/ %
Regular clones	
Mut^S^	347/41
Mut^+^	308/36
Combined	655/77
Irregular clones	
Mut^S^	5/1
Mut^+^	185/22
Combined	190/23
Total	845/100

**Fig. 1:**
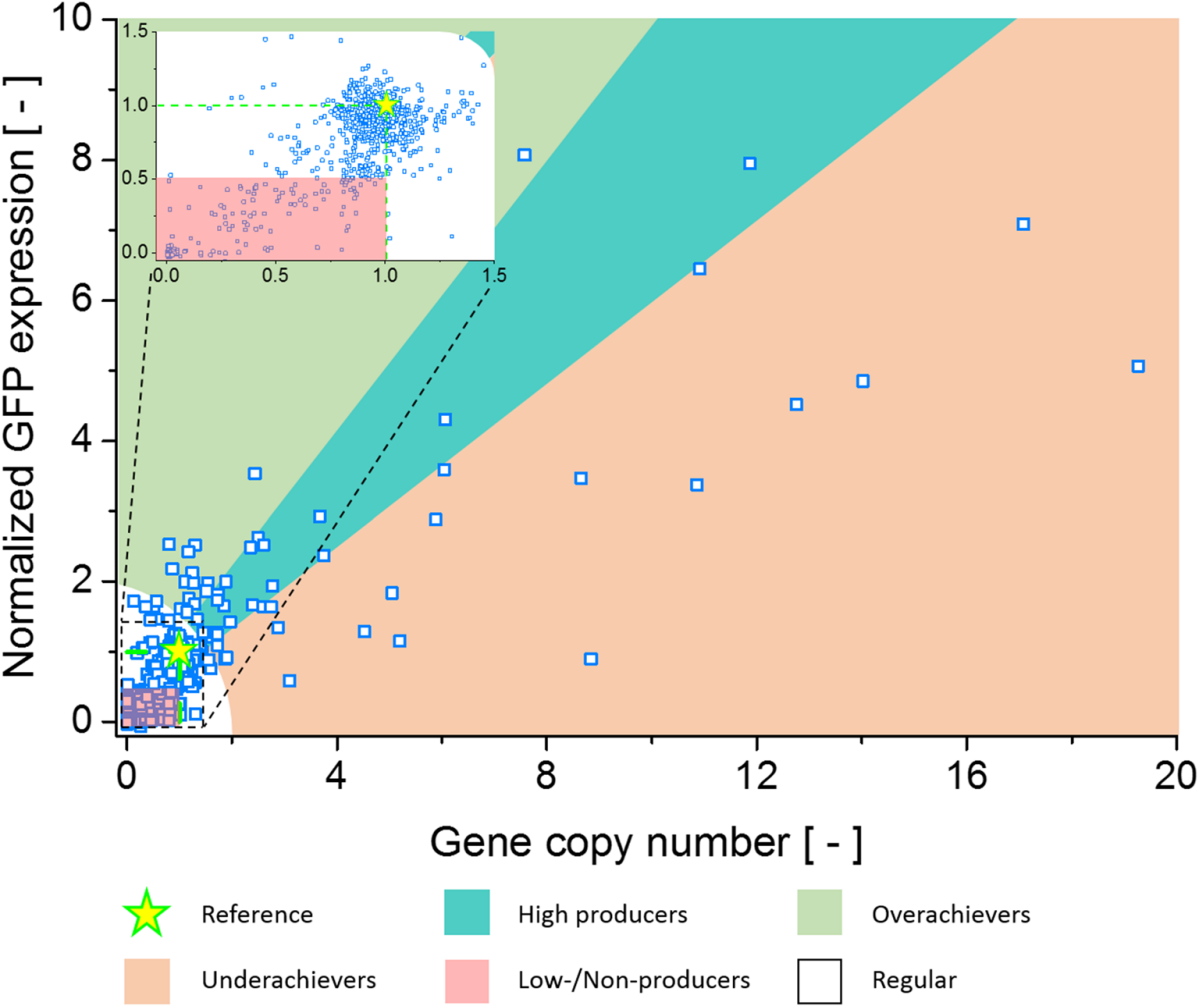
GCN and normalized GFP expression of all 845 characterized *P. pastoris* clones. The reference clone JPS066 with GCN and GFP expression = 1 is shown with a *yellow star*. Areas for clones categorized as high producers, overachievers, underachievers or low-/non-producers (shown in the *cut-out*) are highlighted. Regular clones are found in the *white area*

For a better insight into the diversity of the irregular clones, the relation between GFP expression level and GCN has to be looked at. *P. pastoris* is an industrially important host for recombinant protein expression, therefore these characteristics are at the forefront when it comes to determine whether a clone can meet the requirements of a production process. Interestingly, no clear correlation between GCN and GFP expression level could be seen evaluating all cones (Fig. 1). A wide distribution of clones is visible with no clear pattern. This is in contrast to previously reported results for the relation between GCN and expression level in *P. pastoris* for intracellular protein expression [36]. In other studies, a good linear correlation for intracellular expression was found [37, 38], while secretory expression showcased more complex correlations due to mechanisms like the UPR pathway (unfolded protein response) [34, 39, 40].

To facilitate a clearer understanding of the different kinds of irregular features, the clones were grouped. As shown in Fig. 1 irregular clones could be identified and grouped based on their GCN and GFP expression values. Strains that displayed high GFP expression levels that is paired with a high GCN, two very desirable features for a production strain, were categorized as “high producers”. On the other hand, clones that showed a distinct discrepancy between their GCN and GFP expression were designated “over-“or “underachievers”. Characteristic for these strains is an expression level markedly exceeding (overachiever) or falling below (underachiever) the expression level expected based on the GCN. Additionally, strains with an expression level or GCN notably below 1 or even at 0 were considered “low-/non-producers” making up the group least desirable as a production strain. Table 2 lists all groups the 190 irregular clones were divided into and the basic characteristics of each group. In Additional file 1: Table S1 the concrete criteria used for dividing clones into these groups can be found. By dividing the strains into groups with similar features, a strategy to identify clones of interest for genome sequencing could be conceived.

**Table 2:** Grouping of all 190 irregular clones based on shared properties

Group	No. of clones	Characteristics
High producers	29	High GCN and high expression
Overachievers	21	Low GCN and high expression
Underachievers	40	High GCN and low expression
Low-/non-producers	100	Low/no GCN and expression

### Selection of clones for sequencing and general sequencing results

Using a scoring system (Additional file 1: Table S1), the clones with the most outstanding characteristics from the various groups of irregular clones were selected for genome sequencing. In summary, the scoring system emphasized high deviations from the expected GCN or expression level of 1 and an expression level that did not correlate with the GCN. Based on the scoring system, 31 clones were submitted to genome sequencing. Of these 29 had the Mut^+^ and 2 the Mut^S^ phenotype.

The sequencing runs (2 × 300 bp) on the Illumina MiSeq platform resulted in 80,608,298 reads comprising 24.2 Gb of sequence information. De novo assemblies for each sample generated an average of 37 scaffolds, 76 scaffolded contigs and a size of 9.35 Mb. This represents an average sequence coverage of 83-fold. The average GC content (41.1 %) is in accordance with the findings of Küberl et al. [23], who reported the first genome of *P. pastoris* CBS 7435. Detailed sequencing statistics for each strain can be found in Additional file 2: Table S2.

**Fig. 2:**
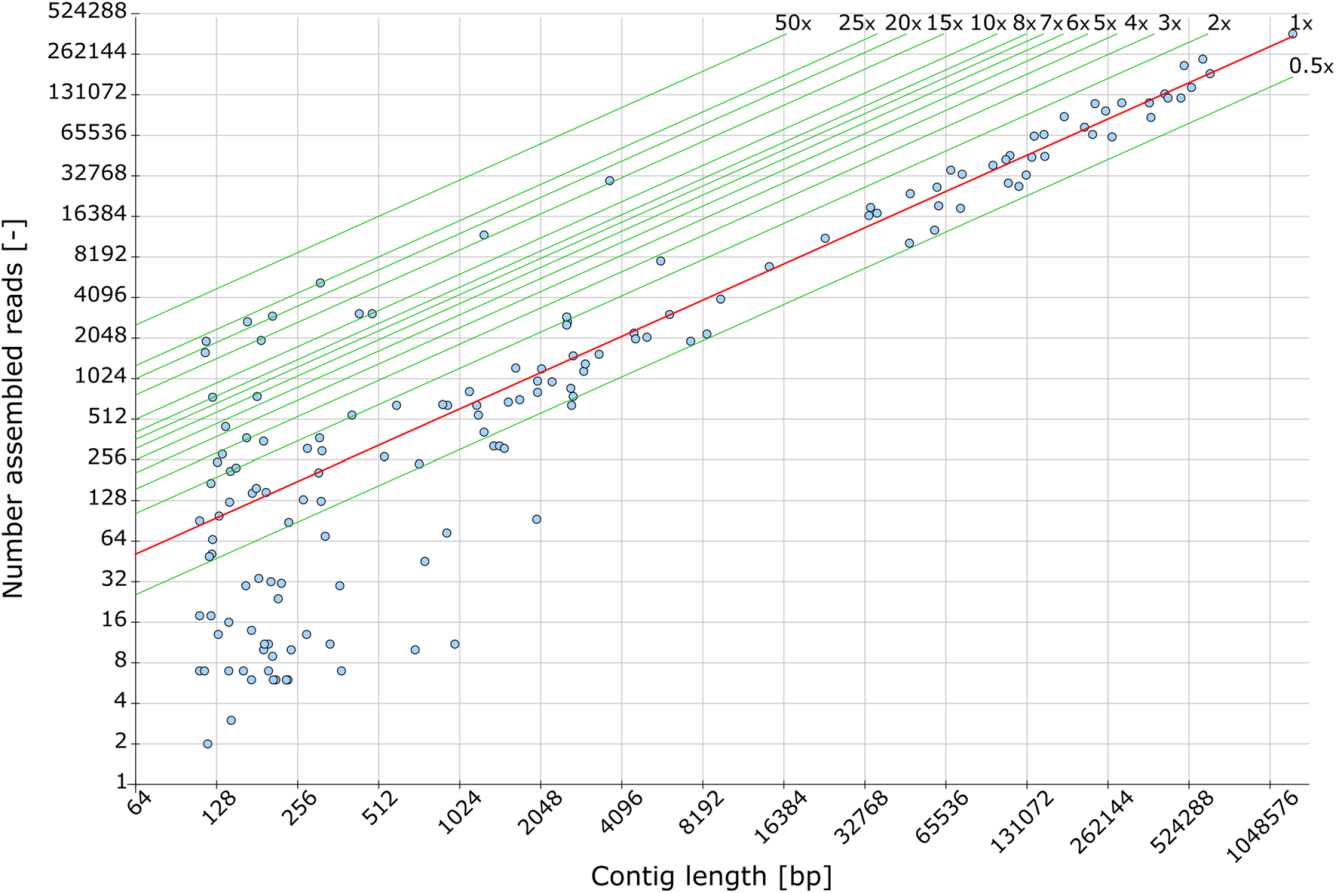
“Contig length vs. read-count” plot for the Low-producer strain JPS379 (EMBL FBTF01000000) contigs in log_2_ scale. *Dots* represent the length of a contig plotted against the number of reads assembled into that contig. The *lines* denote the different predicted coverage ranges within the genome. Underrepresented contigs (group I) are found at a coverage of 0.5 or lower and indicate a secondary isolate in the sequenced sample. Unique contigs are represented by the* red line*, indicating a onefold coverage. Together with the twofold coverage line, they account for group II contigs which make up the chromosome of the main isolate. Above the twofold coverage line contigs belonging to group III can be found. They represent more abundant DNA elements, e.g. mitochondrial DNA

After the sequencing and assembly phase, a “contig-length vs. read-count” plot analysis was performed gaining deeper insights into the composition of the 31 samples. Figure 2 shows an example for one of the Low-producer genomes. In general, assembled contigs can be classified in three different groups. Group I contigs (lower 0.5×) represent low amounts of additional *P. pastoris* isolates in the sample, with small changes in comparison to the main isolate identified by a BLAST approach. The subpopulations of group I, essentially represent mixed-cultures. These sometimes contained low amounts of untransformed *P. pastoris* CBS 7435 (Δ*HIS4*) cells that presumably were supplied with l-histidine from the transformed cells. Contigs of group II (0.5 × to 2×) represent the almost complete chromosomal genome. The contigs of groups III (above 2×) were mostly allocated to the more abundant mitochondrial DNA and the most abundant DNA encoding ribosomal RNAs (rRNA) or other repetitive elements.

While the majority of sequenced clones contained no subpopulation, the occurrence of mixed-cultures with e.g. untransformed cells could have been reduced or completely eliminated by performing dilution plating experiments with transformed strains. If the mixed-cultures were the result of two or more cells adhering to each other after transformation they could form a single colony on the plate, containing both cell types. Dilution plating on suitable plates should separate such mixtures, whereby untransformed cells would be removed. The sequencing results, and especially the “contig-length vs. read-count” analysis, emphasize the necessity of this procedure for *P. pastoris* experiments. Using the high accuracy and sequencing depth of next generation sequencing (NGS) even small contaminations (<5 %) can be identified and culture heterogeneity better understood.

Based on a BLAST approach, the expression cassette was identified in 26 strains, but five strains lacked the *gfp* gene. The amount of contigs for each GFP cassette varies between five and seven contigs, whereas JPS495 stands out with 10 contigs for the cassette. Via in silico finishing, all gaps between cassette contigs were closed. To this end, reads protruding contig ends were used to identify contigs that flank a certain source contig and were used to close the gaps between these contigs by applying CONSED [41]. Based on these results, further analysis was performed. By applying the “contig-length vs. read-count” plot analysis, the different amounts of inserted vector cassettes were calculated. Proportions between one inserted copy and about 20 copies were determined, as described in the methods section. Applying in silico finishing, four linear plasmids were closed and analyzed. Linear plasmids were identified by their left and right end. No additional reads were found that overlap at that position, whereas the coverage of the end contigs were often overrepresented in comparison to the chromosome. No connection to the chromosome and no chromosomal insertion side could be identified, therefore this sequences should represent linear plasmids. Possibly the linear plasmids are the result of expression cassettes being “looped out” from tandem arrays, resulting in instable GCN values for high copy *P. pastoris* clones [42], as illustrated in Additional file 3: Figure S2. Mainly such plasmids contain the vector cassette flanked by parts originating from the *E. coli* backbone of pAHBgl-GFP. The identified plasmids have a size of about 7–9 kb and include only 1–2 more genes on the non-vector cassette parts. On the other hand, it remains unclear how these linear plasmids would withstand degradation or segregational loss. Therefore more experiments are necessary to confirm their existence. In the following sections the various discovered integration events in sequenced *P. pastoris* strains are further discussed and the influence they might have on productivity analyzed.

### Correlation between sequencing and experimental results

**Fig. 3:**
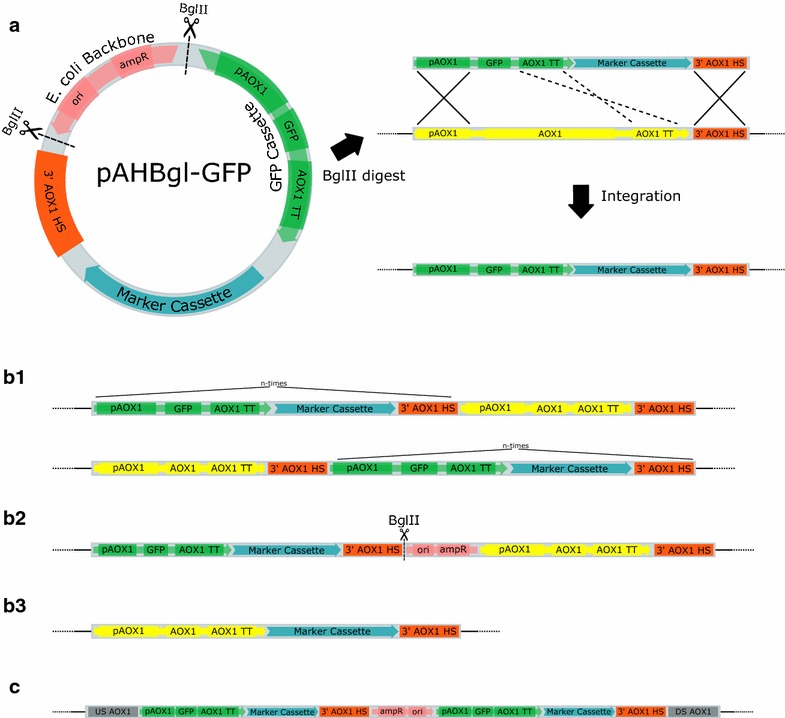
Overview of predicted and observed integration events in *P. pastoris*. **a** The plasmid pAHBgl-GFP is restricted with *Bgl*II, excising the expression cassette containing the *gfp* gene and a selection marker. The *AOX1* promoter (p*AOX1*) and a homology sequence downstream of *AOX1* (3′ *AOX1* HS) facilitate a replacement of the chromosomal *AOX1* locus with the expression cassette via double crossing over (*solid crossed lines*). After homologous recombination the expression cassette is stably integrated into the genome. **b1** Via single cross over the expression cassette is inserted up- or downstream of the *AOX1* locus. This event can occur multiple times, leading to tandem integrations. Other additive integrations can also happen multiple times up- or downstream of *AOX1.* **b2** Additive integration of the whole pAHBgl-GFP plasmid at the *AOX1* locus with highlighted *Bgl*II site. **b3** Replacement of a region downstream of AOX1 with only the selection marker. The event is facilitated by a double crossing over event using 3′ *AOX1* HS and *AOX1* TT (*dashed crossed lines in* **a**). **c** Inversion of the *E. coli* backbone in comparison to the adjacent expression cassettes in the Mut^S^ clone JPS233

Using the data obtained from the sequenced *P. pastoris* clones multiple integration events were discovered (Fig. 3). In addition to the expected replacement of *AOX1* with the GFP cassette (Fig. 3a) the most common event were additional insertions of the expression cassette up- or downstream of the *AOX1* locus (Fig. 3b1). Often not only the cassette, but the complete pAHBgl–GFP vector, was found integrated into the genome hinting at either incomplete digestion or in vivo re-annealing of previously separated restriction fragments (Fig. 3b2). The in vivo re-annealing of fragments after digestion and prior to integration into the chromosome is supported by the orientation of the *E. coli* backbone found in e.g. the Mut^S^ clone JPS233 (Fig. 3c); *EMBL FBTV01000000*). Here, the elements of the *E. coli* backbone are inverted in comparison to the original vector (Fig. 3a), while the adjacent expression cassettes have the same orientation as located on the plasmid. The observed organization cannot be the result of an incompletely digested vector, but rather was caused by *P. pastoris* ligating an inversed *E. coli* backbone to an expression cassette prior to integration. A gel purification step after plasmid digestion and prior to transformation would prevent the in vivo relegation between expression cassette and *E. coli* backbone.

**Fig. 4:**
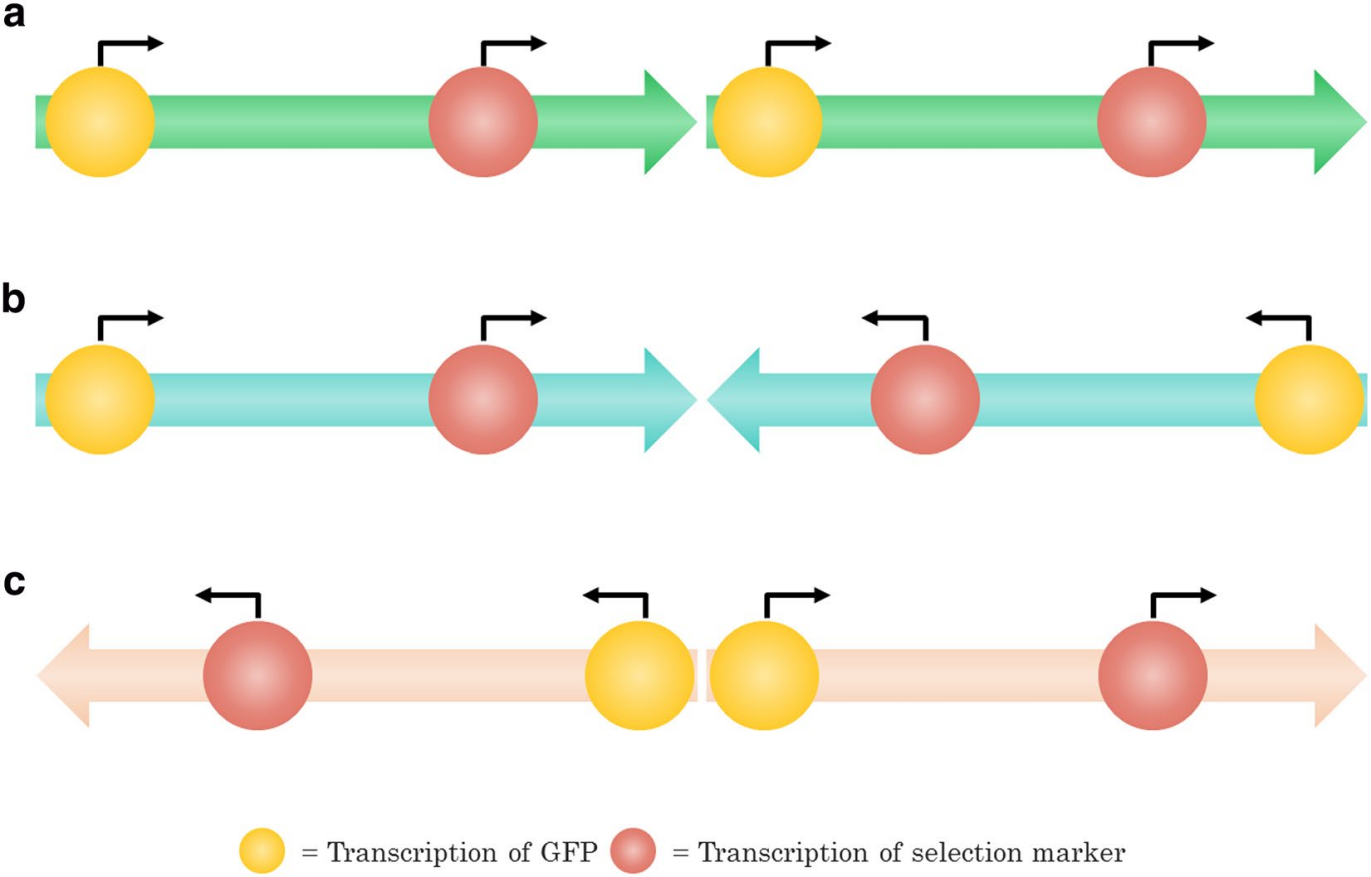
Illustration of the different cassette-to-cassette orientations of adjacent expression cassettes and the transcription start sites found on them. Cassettes are shown in the (**a**) head-to-tail, (**b**) tail-to-tail and (**c**) head-to-head organization

Multiple tandem-integrations of the cassette or the complete vector were found. This is the first genome-sequence based evidence for the prevalence of tandem-integrations as the genetic organization in multi-copy clones of *P. pastoris*, first postulated by Clare et al. [43]. No dispersed distribution of cassettes in multiple different loci on the chromosomes was discovered in the analyzed multi-copy strains. In the majority of sequenced clones with multiple tandem integrations, the head-to-tail order of cassettes was the only organization found. Tail-to-tail or head-to-head sequences were only present in five of the sequenced strains, and in all but one they occurred in equal quantities. The different cassette orders are shown in Fig. 4. Collectively, in 86 cases head-to-tail was encountered, tail-to-tail 14 times and head-to-head 15 times. The exclusivity of head-to-tail in comparison to the inclusivity of head-to-head with tail-to-tail hints at an “either/or” relationship between two main integration mechanisms in *P. pastoris*. Head-to-tail tandem insertions likely integrated via the mechanism described by Clare et al. [43]. A different path of integration was probably responsible for head-to-head and tail-to-tail insertions. Multiple adjacent head-to-head and tail-to-tail integrations would culminate in the observed equilibrium between both arrangements. They could be the result of consecutive integrations of singular expression cassettes, each one using the previous one has homology sequence for additive integration. Only in strain JPS300 (*EMBL FBTO01000000*), head-to-tail as well as head-to-head was found. How these paths differ and why they are seemingly exclusive to one another remains unclear. However, in the following sections the observed correlation between organization of cassettes and their GFP expression are discussed. Furthermore, other findings of the genome sequencing data are correlated with the experimental results for the different groups of irregular clones.

### High producers

**Fig. 5:**
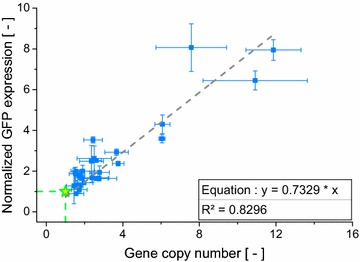
Experimental values for GCN and GFP expression as well as linear regression for clones belonging to the high producers. The reference clone JPS066 is shown as a *green star*. *Error bars* display the standard deviation, with n = 3 × 3

Strains in the high producer group displayed a markedly higher GCN and expression level than the reference clone. Multi-copy clones with up to 12 (±1.4) copies of the *gfp* gene and expression levels up to eightfold (±1.2) higher than the reference clone are present in this group. In addition, a linear correlation between GCN and GFP expression was apparent. In Fig. 5, a good agreement with R^2^ = 0.83 between experimental data is shown. The correlation found here is in accordance with previous reports for the relation between GCN and intracellular protein expression in *P. pastoris* [37, 38]. Notably, the slope of the regression line (0.73) is below 1. At a slope of 1 two copies would produce twice the amount of GFP. Especially clones with a high copy number are likely the cause of this lower incline. High copy clones put more stress on the protein synthesis apparatus of the cell, thereby diminishing the productivity per GFP cassette and lowering the slope of the linear regression curve.

In total, six clones from this group were selected for genome sequencing. As was expected based on the qPCR experiments multiple copies of the cassette could be detected. Based on the read frequency, up to 15 copies of the *gfp* gene were present. In addition to the GFP cassette, in some cases the *E. coli* backbone of pAHBgl–GFP was additionally inserted in-between two cassettes or directly adjacent to another one. All integration events occurred at the *AOX1* locus, even in Mut^+^ strains. While it can not be excluded that unsequenced high producer clones contain integrations at other loci, it appears no off-target integrations occurred in this group. Since high producers are most likely to be selected for production purposes, the lack of random gene disruptions increases their suitability for industrial applications.

Except clone JPS535 (*EMBL FBTY01000000*), only head-to-tail tandem integrations were found in high producer clones, suggesting that this organization might be beneficial for productivity. For gaining further insight, the differences seen in comparison to over- and underachiever clones in the next section have to be included.

### Over- and underachievers

**Fig. 6:**
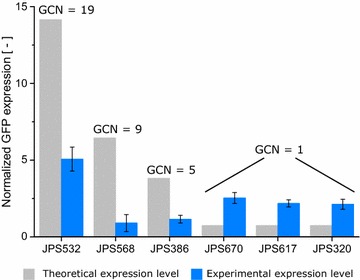
Comparison between theoretical and experimental GFP expression for selected over—(JPS532, JPS568 and JPS386) and underachiever (JPS670, JPS617, JPS320) clones. The theoretical values were calculated using the GCN and the equation shown in Fig. 5. *Error bars* represent the standard deviation, with n = 3 × 3

Individual clones assigned to one of these groups displayed either a markedly higher or lower GFP expression as expected based on the GCN. In total, eight over- and nine underachiever clones were selected for genome sequencing. The discrepancy between experimental and theoretical expression level (calculated using the GCN and the equation shown in Fig. 5) are clearly visible in Fig. 6.

For certain overachiever clones the observed GFP expression was more than twofold increased in comparison to the theoretical value. On the other hand, some underachiever strains showcased expression levels far lower than half the expected GFP expression level. Notably, in three sequenced overachiever clones the abundances of the *gfp* gene calculated from sequence coverage indicate the presence of two copies. These clones would therefore have an expression level in line with their GCN and belong to the group of high producer strains. Multiple explanations are possible as to why the GCN of the affected clones was consistently lower in the qPCR experiments. Potentially, the PCR efficiency in these clones differed too much from the reference, thereby producing inaccurate qPCR predictions. Alternatively, the physical organization of the *gfp* locus in the isolated genomic DNA (gDNA) hindered the annealing of primers during qPCR. Nevertheless, in five sequenced overachiever strains only a single copy of *gfp* was found.

**Fig. 7:**
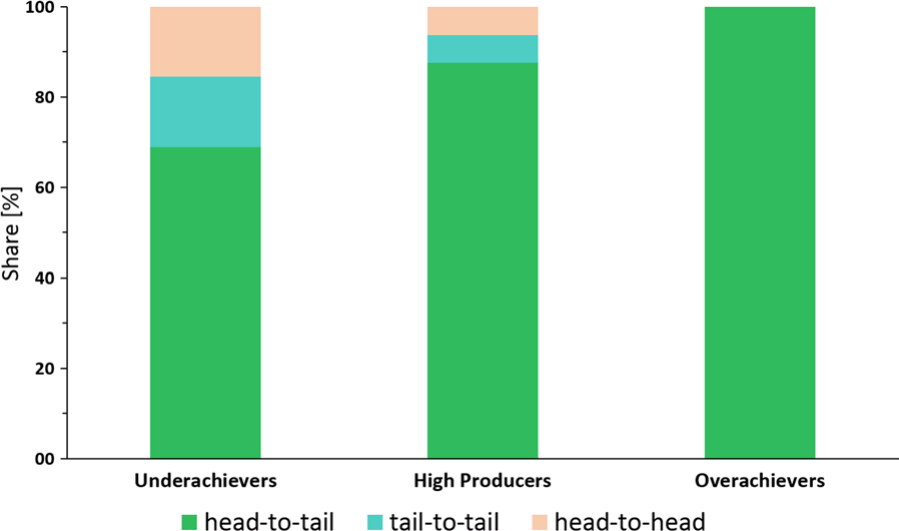
Distribution of different cassette orientations among the sequenced multi-copy clones of the under—and overachievers as well as high producers

For an in-depth evaluation of the cause for deviant production characteristics the orientation of the cassettes to one another was analyzed. In strains of the overachiever group only head-to-tail tandem integrations were found. On the other hand underachiever clones showed the highest proportion of head-to-head and tail-to-tail integrations with 30 % of all tandem integrations. In some underachiever clones a GCN >10 was found. It is possible that in these clones the decreased productivity was due to the high gene dosage triggering cytosolic proteases. The distribution of different cassette orientations among the groups of high producers, as well as under- and overachievers is shown in Fig. 7.

In combination with the findings described for the high producer group a trend is visible. Good production characteristics correlate with a head-to-tail orientation of adjacent cassettes. Head-to-tail organization ensures that all cassettes are read in the same direction. The lower GFP expression of strains with head-to-head and tail-to-tail integrations potentially was due to physical obstructions between RNA polymerases on adjacent cassettes during transcription. Crampton et al. [44] demonstrated via atomic force microscopy the premature stop of RNA polymerases on DNA with convergent promoters due to collision events. For the tail-to-tail orientation a “Head-on collision” of RNA polymerases seems less relevant. In this constellation only the marker cassettes are directly convergent with two 3′ *AOX1* HS (homology sequence downstream of *AOX1*, each 0.7 kb) separating them (Fig. 4b). However, in a head-to-head orientation the p*AOX1* of neighboring cassettes are directly adjacent to one another (Fig. 4c). This could culminate in mutual obstruction of RNA polymerases binding during the initiation of transcription, resulting in lower expression of the target protein.

In consequence, the construction of vectors that contain multiple expression cassettes in the head-to-tail orientation should lead to clones with an on average higher productivity than alternative approaches for the generation of multi-copy clones. Vassileva et al. [38] demonstrated the successful application of this strategy for the production of hepatitis B surface antigen, displaying a good correlation between GCN and product titer. If the transformation technique allows for other tandem integrations of cassettes, especially the head-to-head orientation, clones with a lower productivity are to be expected. This could be an explanation for some of the discrepancies found in other studies between GCN and expression level, if the orientation of expression cassettes was not determined [36].

### Low-/non-producers

Figure 1 displays a large number of clones producing only low amounts or no detectable GFP, the so called low- or non-producers. Six clones belonging to these groups were analyzed via genome sequencing. Many of these strains gave GCN values below 0.5 in qPCR experiments, indicating that a copy of the GFP cassette was present but the copy frequency was lower than that of the calibrator gene. Either the PCR efficiency for the *gfp* target was affected by e.g. the physical configuration of the GFP cassette or the mixed-culture phenomena caused a greater abundance of *ARG4* genes compared to *gfp* genes. With the mixed-culture phenomena the lower production, illustrated with the Low-producer strain JPS379 (*EMBL FBTF01000000*) in Fig. 2, can also be explained. A higher ratio of untransformed cells would imply that more cells in the culture media consume methanol without producing GFP. Thus, while GCN values <1 during qPCR experiments still indicate the presence of a full length expression cassette, they might also be used to identify clones with a lower productivity than is to be expected with GCN = 1. The findings of the sequencing experiments of four low-producer clones support this theory. Cultures of these strains always contained likely untransformed cells, as discovered by “contig-length vs. read-count”-plots discussed earlier.

In contrast to the low-producer clones, non-producer clones exhibited no detectable GFP expression and also no detectable GCN. Since they also grew normally in minimal medium without histidine, they were not untransformed cells. Genome sequencing of the non-producer strains JPS056 and JPS060 (*EMBL FBTH01000000* and *FBVQ01000000*) revealed that they shared the same genotype. Only the marker cassette region of pAHBgl-GFP had integrated in the *AOX1* locus (Fig. 3b3). This insertion was most likely facilitated by a double crossover homologous recombination between the *AOX1* terminator *(AOX1* TT) and the 3′ *AOX1* HS regions on the expression cassette and the *AOX1* chromosomal locus. As shown in Fig. 3a with dashed lines, *AOX1* TT presents a third homology sequence in addition to the two used for replacement of *AOX1*. In total, 68 clones exhibited the same phenotype (GCN and GFP expression level = 0) as JPS056 and JPS060. Therefore, this secondary homologous recombination event appeared to occur quite frequently. As a consequence of the insertion via *AOX1* TT and 3′ *AOX1* HS, an uncharacterized gene downstream of *AOX1*, the 810 bp long *YDR514C*, is disrupted. At the 5′ end of Y*DR514C,* 165 bp are homologous to the *AOX1* TT, while at the 3′ end 509 bp are homologous to 3′ *AOX1* HS. Thus, base 510 to 645 of *YDR514C* are replaced with the marker cassette during this secondary homologous recombination event, which can be seen in the genome sequences of clone JPS056 and JPS060. Using this information, other clones with similar phenotype were analyzed via PCR for the integrity of *YDR514C*. A total of 64 strains with the same genotype as discovered in JPS056 and JPS060 could be identified. Thus, such false-positive clones accounted for approximately 8 % of all clones. They bypass the selection process and unnecessarily increase the workload e.g. in studies in which every clone is of interest. The irregular integration event seemed to depend on the close proximity of *AOX1* TT and 3′ *AOX1* HS on the expression cassette allowing insertion of the marker cassette without the target gene via homologous recombination. Resulting clones can grow in selective media and show the Mut^+^ phenotype, but cannot produce the target gene. For further analysis of this phenomenon a novel vector was constructed and transformed into *P. pastoris*.

### An optimized vector to prevent false-positive clones

Based on the findings for non-producer clones a variant of pAHBgl-GFP was constructed, aiming to prevent the creation of false-positive clones due to the integration of only the marker cassette. Since the erroneous integration event was assumed to be facilitated by a homologous recombination between the *AOX1* TT and 3′ *AOX1* HS regions, they were the key optimization targets. By replacing the terminator and leaving the 3′ *AOX1* HS unchanged, the core functionality of the vector would remain unaltered, thus this strategy was chosen. The *CYC1* (cytochrome c iso-1) terminator (*CYC1* TT) from *S. cerevisiae* was selected as replacement for multiple reasons: It shows no sequence similarities to the genome of *P. pastoris* CBS 7435 and is a well-studied, widely used terminator in *S. cerevisiae* [45, 46]. Additionally, *CYC1* TT has been used before as part of *P. pastoris* vectors, albeit not in combination with p*AOX1* [47, 48]. The resulting vector was named pAHBgl–GFP-CYC.

After transformation with pAHBgl-GFP-CYC, 120 clones were picked and characterized for their GFP expression using the same methods as described above. Their expression level was normalized to the same reference clone JPS066. In all 120 clones GFP expression was detectable. Using the Pearson’s Chi squared test, it was determined that the lack of false-positive clones was significant (Table 3).

**Table 3:** Occurrence of false-positive clones when using pAHBgl-GFP compared to pAHBgl-GFP-CYC

Vector	Total clones	False-positive clones (no./ %)	Χ^2^ value	p value
pAHBgl-GFP	845	64/8	–	–
pAHBgl-GFP-CYC	120	0/0	10.91	0.0001–0.001

The absence of false-positive clones strongly suggests that the integration of the marker cassette found in JPS056 and JPS060 was indeed mediated by a double crossing over event using the *AOX1* TT and 3′ *AOX1* HS regions. Multiple commercial and non-commercial vectors for *P. pastoris* are targeted for *AOX1* replacement, while putting the gene of interest under the control of p*AOX1* and *AOX1* TT [49, 50]. Integration of only the marker cassette by the mechanism described here can potentially also occur using these plasmids. Therefore it would seem advisable to switch to a different terminator for the gene of interest in order to prevent an increased workload for finding the producer strains, due to false-positive clones.

It has to be noted, however, that pAHBgl-GFP-CYC strains produced markedly lower amounts of GFP than pAHBgl-GFP clones on average (Additional file 3: Figure S3). Likely *CYC1* TT is not strong enough as a terminator for the exceptionally strong p*AOX1*. Thereby, faulty transcription termination and inactive gene products occur. For high-level production, a different terminator ought to be used. In a recent study multiple terminators (and other regulatory elements) from *P. pastoris* were characterized, providing a good starting point for finding a more effective replacement terminator [51].

## Conclusion

Multiple unexpected integration events were discovered during genome sequencing and correlated with the production characteristics of the clones. By analyzing the connection between genome sequence and classic characterization experiments, many novel insights were obtained. The findings demonstrate that the combination of both methods enables deeper understanding than using them separately. Previously postulated theories regarding the generation of multi-copy tandem head-to-tail integrations and in vivo ligation events prior to integration could be verified [43]. It was found that the head-to-tail modus is the dominant insertion pathway, markedly outweighing head-to-head and tail-to-tail integrations. Both pathways seem to be exclusive to one another. The data also suggests that head-to-head and tail-to-tail integrations have a negative impact on productivity. A likely cause is the close proximity of p*AOX1* of neighboring head-to-head cassettes. As a result RNA polymerases obstruct each other during transcription. Therefore it seems advisable to use methods specifically generating head-to-tail multi-copy clones, if aiming to increase the product titer via the gene dosage.

In some sequenced strains the presence of multiple genotypes in the form of a mixed-culture was observed, sometimes containing untransformed cells likely provided with l-histidine by transformed cells. Using dilution plating procedures after transformation should eliminate most, if not all, of the mixed-cultures containing untransformed cells. Employing antibiotics like Zeocin for selection ought to reduce the risk of such contaminations as well. The discovery of these subpopulations via genome sequencing supports the validity of the dilution plating procedure, often used in experiments involving yeast.

A secondary double crossing over event using *AOX1* TT and the 3′ *AOX1* HS led to the integration of only the marker cassette and the creation of false positive clones in about 8 % of all clones. Such clones result in an increased workload when assaying transformed cells for their productivity. By replacing *AOX1* TT with the non-homologous *CYC1* TT, we could show that no more false-positive clones occurred after transformation. Thereby underlining the validity of theories derived from correlating experimental and genome sequencing results. However, productivity was markedly lower, likely caused by inefficient transcription termination, suggesting that a more suitable terminator needs to be implemented.

Notably, the expression cassette was always found at the *AOX1* locus in the analyzed clones, for both Mut^S^ and Mut^+^ strains. Especially for high producers, the best suited strains for industrial applications, the apparent absence of integrations at other loci is desirable. Random integrations as a result of NHEJ have been described for *P. pastoris*. In the present study a vector system was used, designed to prevent off-target integration [5]. Additionally, the selection process for sequencing emphasized productivity characteristics. It is therefore possible that random integrations at other loci were overlooked as they had no or only a small impact on the expression of GFP. The majority of clones had a GCN and expression level of ca. 1. Potentially, many of these clones harbor an expression cassette integrated at a locus other than *AOX1*, which did not affect productivity.

## Methods

### Microorganisms and cultivation conditions

*Escherichia coli* KRX (Promega, USA) was used for plasmid construction and propagation work. KRX was cultivated in LB (Lysogeny Broth) medium supplemented with 100 µg/mL ampicillin. For experiments involving *P. pastoris* CBS 7435 (Δ*HIS4*), obtained from ACIB (Austrian Center of Industrial Biotechnology, Austria) as well as the wild type CBS 7435 (CECT 11047 at Spanish Type Culture Collection, Spain), were used. *Saccharomyces cerevisiae* wild type strain LBG H620 was provided by the Institute for Agricultural Bacteriology and Fermentation Biology, ETH Zurich, Switzerland. Yeast shake flask cultivations were carried out in BMD (Buffered Minimal Dextrose) [49] or YPD (Yeast Peptone Dextrose) medium, supplemented with 4 mg/L l-histidine when necessary. Experiments in 96-deep-well plates with 2.4 mL total volume (Eppendorf, Germany) used BMD, BMM2 (Buffered Minimal Methanol) and BMM10 as previously described by Weis et al. [30] and Hartner et al. [31]. In brief, BMD is used for the growth phase while BMM2 and BMM10 induce expression of the target gene by maintaining a 0.5 % (*v*/*v*) methanol content in the culture medium. The 96-deep-well plates contained up to 500 µL of culture media, were sealed with sterile Breathseal film (Greiner, Germany) and were shaken at 340 rpm at 28 °C.

### Plasmid construction and transformation

Primers were designed using SnapGene (GSL Biotech, USA). The sequences of all primers used in this study can be found in Additional file 4: Table S3. In order to construct a vector for intracellular expression of GFP*uv* in *P. pastoris*, the plasmid pAHBgl from ACIB, Austria was used as the basis vector. pAHBgl allows for selection based on complementation of the histidine auxotrophy, lacks a secretion signal and can be used for ends-out insertion via linearization with *Bgl*II prior to transformation [5]. Using the Gibson assembly technique [52], the *gfpuv* gene was amplified via PCR from the plasmid pBAD-GFP*uv* [29] and inserted into linearized pAHBgl, resulting in pAHBgl–GFP. Similarly the *AOX1* TT of pAHBgl–GFP was replaced by means of Gibson assembly with the *CYC1* TT amplified from gDNA of *S. cerevisiae* LBG H620, creating pAHBgl-GFP-CYC.

pAHBgl-GFP and pAHBgl-GFP-CYC were amplified in *E. coli* KRX. For transformation into *P. pastoris* CBS 7435 (Δ*HIS4*) the plasmids were extracted with the Wizard^®^ Plus SV Minipreps DNA Purification System (Promega, USA). Purified plasmids were digested with *Bgl*II to facilitate ends-out insertion of the GFP expression cassette targeted for replacement of the native *AOX1*. *P. pastoris* CBS 7435 (Δ*HIS4*) was transformed according to Wu and Letchworth [53], with 2–3 µg of digested plasmid DNA per transformation. Cells were spread immediately after transformation onto MD (Minimal Dextrose) plates [49] and incubated at 28 °C for 3–4 days before picking clones. The transformants were both used for the following characterization experiments and stored at −80 °C in 12.5 % (*w*/*v*) glycerol to serve as a master strain bank for later analysis.

### Characterization of *P. pastoris* clones

For determination of the Mut-phenotype the plating test as described in the EasySelect™ *Pichia* Expression Kit Manual (Invitrogen, USA) was used, employing MD and MM (minimal methanol) plates [49].

GFP expression was assayed in a 96-deep-well plate format using established protocols for *P. pastoris* [30, 31]. Clones were always cultivated in triplicate per plate, with clones belonging to high interest groups being cultivated on two additional deep-well plates. In order to normalize the GFP expression values of each clone, independent of the plate and experiment batch a reference clone had to be selected. The reference clone (JPS066) is a Mut^S^, single-copy clone chosen from among the first 100 clones in a preliminary experiment. JPS066 exhibited a GFP fluorescence level closest to the mean of all single-copy clones of that test. Therefore the GFP fluorescence per OD_600_ of the reference clone was set as 1 and all other clones normalized to it. In the following experiments the reference clone as well as the untransformed CBS 7435 (Δ*HIS4*) were always cultivated on each deep-well plate for normalization. Eq. (1) was used for calculating the GFP expression level of each strain. Both GFP fluorescence (excitation 390 nm, emission 510 nm) and OD_600_ were measured using a SPECTRAFluor Plus microplate reader (Tecan, Switzerland). The value for the GFP expression level in the results and discussion section always represents the normalized expression level 60 h after the start of the methanol induction.

1 $$GFP_{X} = \frac{{\frac{{RFU_{X} - RFU_{B} }}{{OD_{X} - OD_{B} }}}}{{\frac{{RFU_{R} - RFU_{B} }}{{OD_{R} - OD_{B} }}}}$$

where GFP_X_ is the normalized GFP expression level of clone X, RFU_X/R/B_ the relative fluorescence units of GFP for clone X, the reference clone R or the blank [CBS 7435 (Δ*HIS4*)] and OD_X/R/B_ is the OD_600_ value of clone X, the reference clone R or the blank (medium), respectively.

gDNA was extracted from *P. pastoris* using the MasterPure™ Yeast DNA Purification Kit (Epicentre, USA). The GCN was determined in qPCR experiments in technical duplicates according to previously reported methods [32] using the Rotor-Gene SYBR^®^ Green PCR Kit (Qiagen, Germany) and a LightCycler^®^ 96 system (Roche, Switzerland). Irregular clones were assayed in two additional biological replicates, each with technical duplicates. In brief, the GCN of the *gfp* gene was assayed using relative quantification based on the 2^−ΔΔCt^ method [54]. The single copy gene *ARG4* was chosen as calibrator gene. The primers designed for qPCR exhibited similar T_M_-values (59 ± 1 °C) and amplicon sizes (100 ± 1 bp). Their nucleotide sequences can be found in Additional file 4: Table S3. Using gDNA from the reference clone JPS066 in quadruple determination over 3.5 logs of copy quantity, a calibration curve for the *ARG4* and *gfp* target was created to determine the PCR-efficiencies for each target (Additional file 3: Figure S1). These PCR-Efficiencies were used for the following assays. Approximately 1 ng gDNA was used per qPCR reaction.

### Sequencing of 31 selected *P. pastoris* strains and genome assembly

gDNA of 31 selected *P. pastoris* strains was isolated for high throughput sequencing as described above. The quality of the DNA was assessed by gel-electrophoresis and the quantity was estimated by a fluorescence-based method using the Quant-iT PicoGreen dsDNA kit (Invitrogen, USA) and the Tecan Infinite 200 Microplate Reader (Tecan, Switzerland).

For sequencing of the *P. pastoris* strains, paired-end sequencing libraries (TruSeq sample preparation kit; Illumina, USA) were constructed according to the manufacturer’s protocol. The genome sequences of the *P. pastoris* strains were established on the Illumina MiSeq system by two paired-end sequencing runs (2 × 300 bp) with a distance range of about 500 bp. Upon sequencing and processing of the raw data, *de novo* assemblies were performed using the GS *De Novo* Assembler, software release version 2.8. (Roche, Switzerland) with default settings. The assembled draft sequences for the *P. pastoris* genomes were deposited in the EMBL-EBI database under the study id *PRJEB12220*.

### Bioinformatic analyses of the 31 Pichia pastoris strains

First insights into the quality of the assemblies were provided by a “Contig-length vs. read-count plot” analysis [55–57]. In the plot the length of each contig (x-axis) is assessed against the number of assembled reads from the corresponding contig (y-axis). The values of lines representing the contig coverage were calculated from the contig length and the number of assembled reads of contigs longer than 10,000 bases, because the probability for these is higher than that for shorter contigs. The contig length distribution covers several orders of magnitude, so a double logarithmic scale was chosen for the axes. Both axes are logarithmic to the base two. This way even very long contigs can be presented clearly. For the calculation of the coverage lines also a double-logarithmic form was used.

Using the readcount and length values the relative abundance of contigs (e.g. the expression cassette) was calculated. With Eq. (2) and the median raw coverage (C) of all contigs longer than 10,000 bp the normalized coverage of a contig [cov(c_i_)] can be determined. Contigs longer than 10,000 bp are expected to have a single fold coverage.

2 $$cov(c_{i} ) = \frac{{readcount(c_{i} )}}{{length(c_{i} ) \cdot C}}$$

cov(c_i_) values between 0.5 and 1.5 indicate that the corresponding contigs are represented once in the genome; ratios lower than 0.5 indicate underrepresented contigs and ratios higher 1.5 indicate overrepresented contigs (e.g. a multi-copy clone).

For more insight, genomic contigs resulting after the assembly were analyzed for large local similarities applying the BLASTn algorithm [58]. Each contig was compared to a local database including the pAHBgl-GFP vector sequence. Hits with an e-value >1 *×* 10^−20^ and a sequence identity of 100 % were analyzed in detail. In the first step, an in silico based finishing approach was used to close the gaps of vector sequence [23, 59, 60]. In the next step, the raw sequence coverage of the vector sequence was calculated and normalized based on “contig-length vs. read-count plot” analysis to detect the amount of inserted units. In addition, using the in silico based finishing approach the insertion site of each vector into the *P. pastoris* genome was identified. Annotation of relevant contigs from sequenced clones was done using SnapGene (GSL Biotech, USA).

## Additional files

10.1186/s12934-016-0486-7Criteria used for sorting *P. pastoris* strains into different groups of “regular” and “irregular” clones, as well as scoring system for selection of clones for sequencing.
10.1186/s12934-016-0486-7Detailed genome sequencing statistics for all sequenced clones.
10.1186/s12934-016-0486-7Additional Figure 1 (qPCR calibration curve), Figure 2 (looping-out events) and Figure 3 (Comparison of GFP expression between pAHBgl-GFP and pAHBgl-GFP-CYC clones).
10.1186/s12934-016-0486-7Primer sequences.

## Abbreviations

*AOX1/2*: alcohol oxidase 1/2
p*AOX1*: alcohol oxidase 1 promoter
*AOX1* TT: alcohol oxidase 1 transcription terminator
3′ *AOX1* HS: homology sequence downstream of *AOX1*
BMD: buffered minimal dextrose
BMM: buffered minimal methanol
*CYC1* TT: cytochrome c iso-1 transcription terminator
GCN: gene copy number
gDNA: genomic DNA
LB: lysogeny broth
MD: minimal dextrose
MM: minimal methanol
Mut^S/+^: methanol utilization slow/plus
NHEJ: non-homologous end-joining
YPD: yeast peptone dextrose
