# Supplementary material for: Integration event induced changes in recombinant protein productivity in Pichia pastoris discovered by whole genome sequencing and derived vector optimization
Source: Microb Cell Fact. 2016 May 20;15:84. doi: 10.1186/s12934-016-0486-7 (PMC4874018; doi:10.1186/s12934-016-0486-7)
Supplement: Supplementary file 3 — 10.1186/s12934-016-0486-7Additional Figure 1 (qPCR calibration curve), Figure 2 (looping-out events) and Figure 3 (Comparison of GFP expression between pAHBgl-GFP and pAHBgl-GFP-CYC clones). [file 12934_2016_486_MOESM3_ESM.doc]

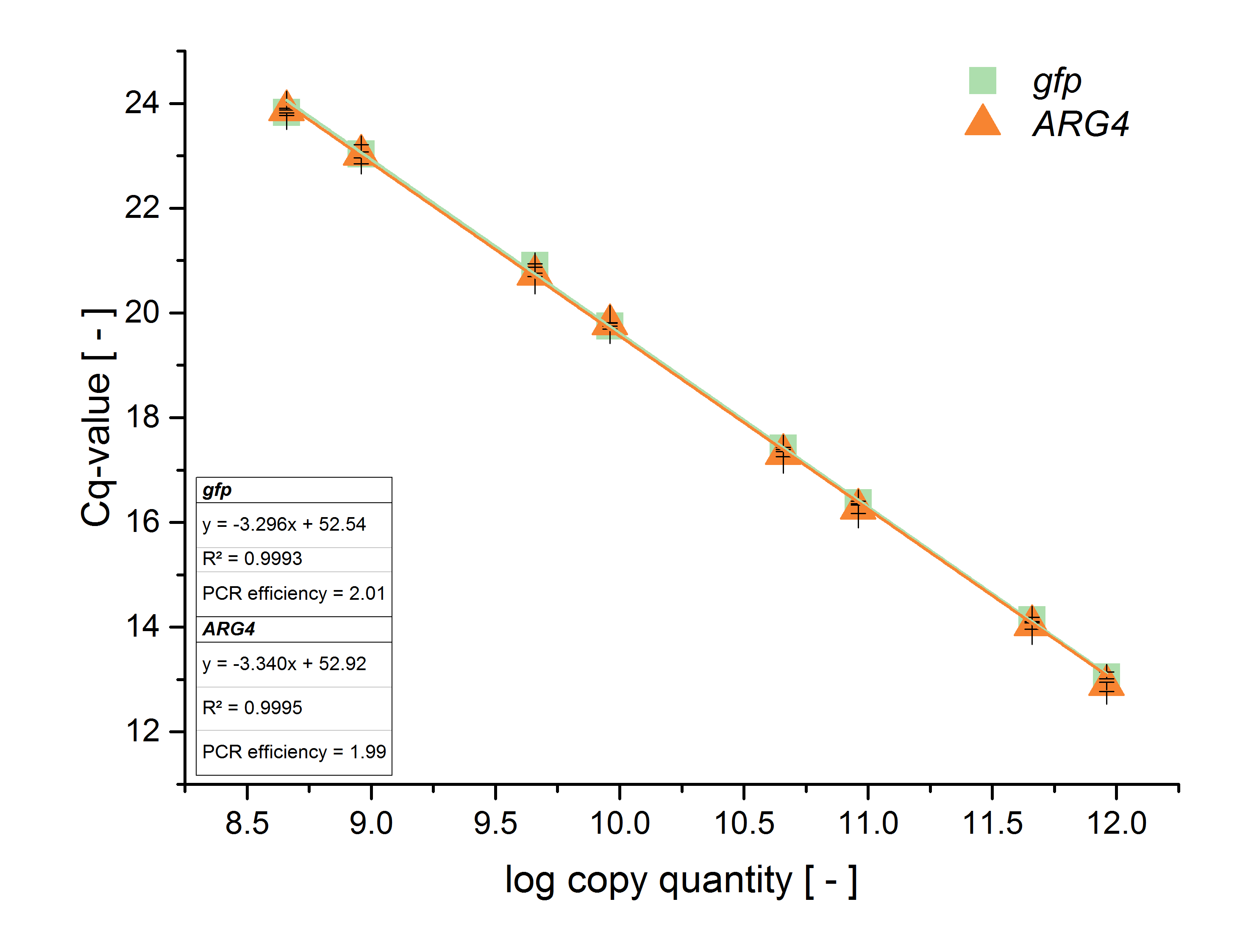


Fig. S1: qPCR calibration curve for the target (gfp) and the calibrator gene (ARG4). gDNA from the reference strain JPS066 was used as a template. The equation for the linear fit, R²- and PCR efficiency values for both gfp and ARG4 are shown in the integrated table. Error bars represent the standard deviation with n = 4


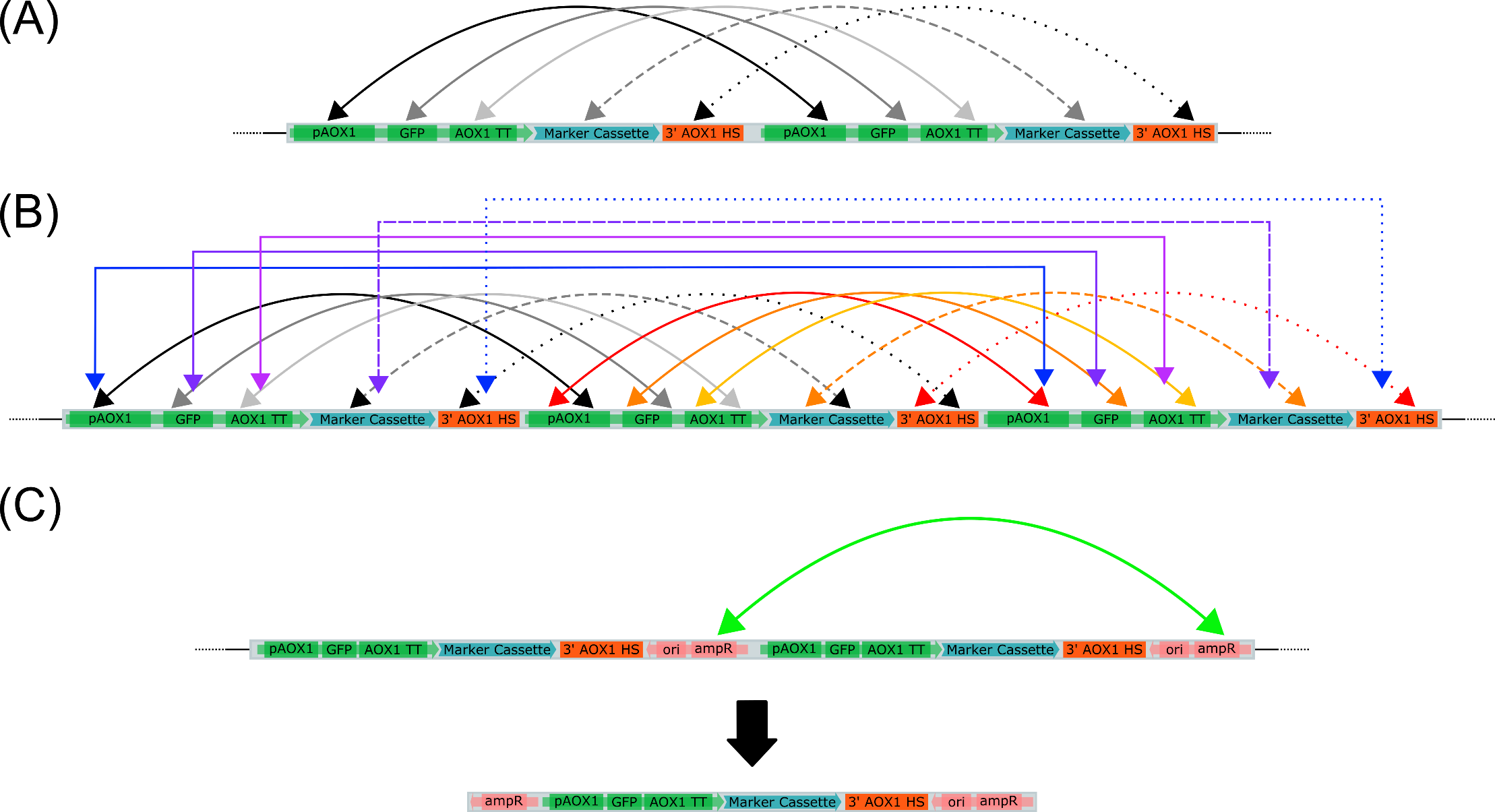


Fig. S2: Illustration of possible looping out recombination events between adjacent expression cassettes based on the vector design and a head-to-tail integration pattern. **(A)** Potential recombination events between two adjacent expression cassettes. **(B)** With three neighboring cassettes the amount of possible recombination events is increased threefold, compared to two cassettes. **(C)** Example loop out recombination and the resulting linear plasmid. The depicted reaction is based on the linear plasmid found during genome sequencing of strain JPS094 (**EMBL FBTE01000000**).


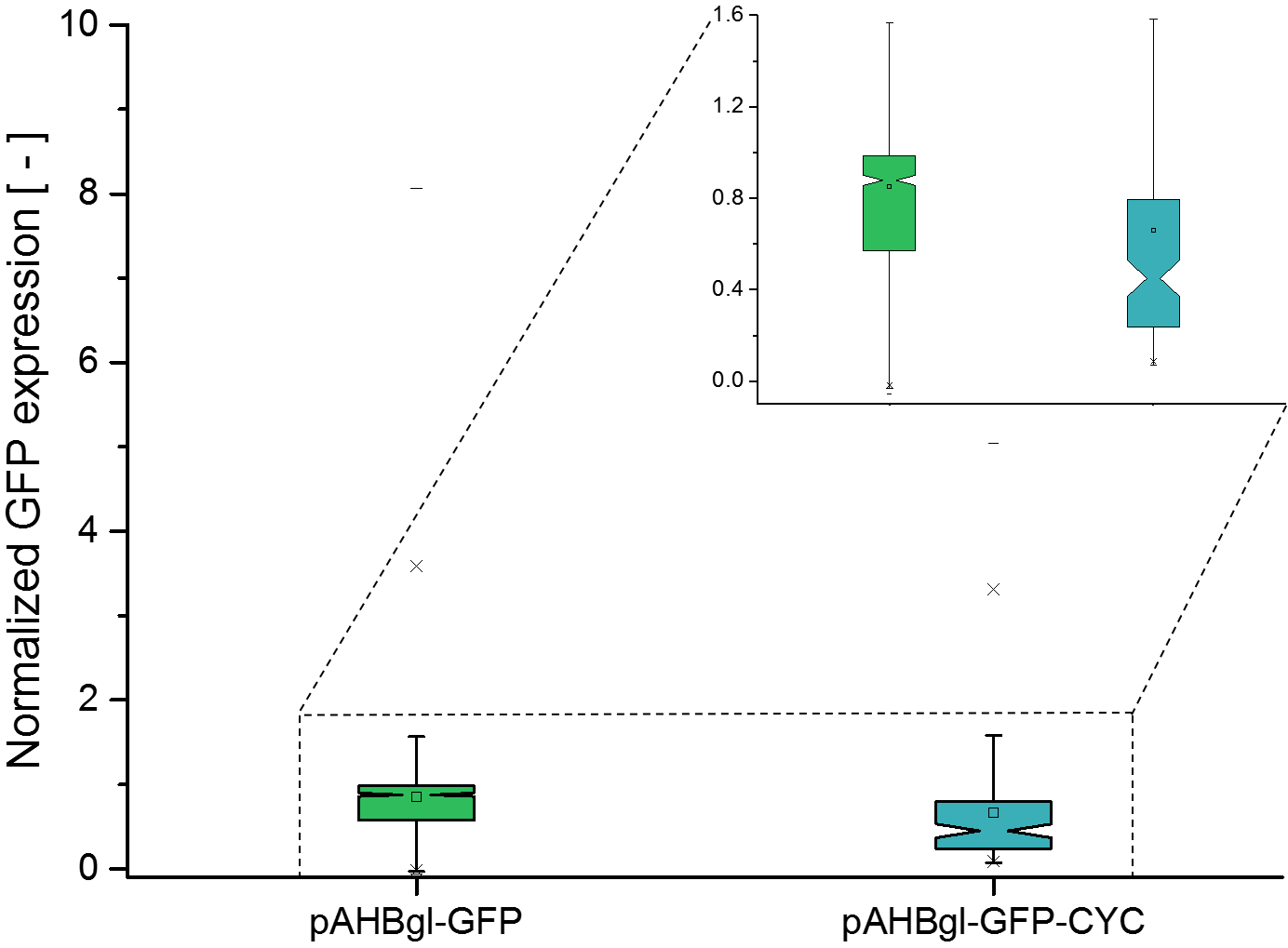


Fig. S3: Comparison of the normalized GFP expression of P. pastoris clones transformed either with pAHBgl-GFP or pAHBgl-GFP-CYC. 845 paHBgl-GFP and 120 pAHBgl-GFP-CYC strains were analyzed. The cut-out highlights the on-average lower expression level for clones transformed with pAHBgl-GFP-CYC in comparison to the original vector.
